# Supplementary material for: Family Resilience and Dyadic Coping during the Outbreak of the COVID-19 Pandemic in Italy: Their Protective Role in Hedonic and Eudaimonic Well-Being
Source: Int J Environ Res Public Health. 2023 Sep 6;20(18):6719. doi: 10.3390/ijerph20186719 (PMC10530876; doi:10.3390/ijerph20186719)
Supplement: Supplementary file 1 [file ijerph-20-06719-s001.zip › ijerph-2517428-supplementary.pdf]

Supplementary materials

Table S1. Partially standardized regression coefficients and percentage of variance explained for joint moderation effects of Gender and Employment status on the association between Family resilience and PGWBI dimensions – Study 1.

Moderator		Anxiety	Depressed mood	Positive well-being	Self-control	Vitality
Males	Full-time workers	-0.31*	-0.09	-0.08	-0.22	-0.01
	Part-time workers	-0.24	0.19	0.05	0.15	-0.47*
	Not working students	-0.23	-0.07	-0.03	-0.10	-0.12
Females	Full-time	0.08	0.22	0.21	-0.01	0.00
	Part-time	0.36**	0.50***	0.34**	36**	0.40***
	Not working students	0.09	0.23**	0.26**	0.11	0.28**
	Interaction coefficient for gender	*0.40**	*0.31°*	*0.29°*	*0.21*	*0.22*
	Interaction coefficients for employment status (part-time workers vs others)	*0.28*	*0.28*	*0.13*	*0.37**	*0.32°*
	Interaction coefficients for employment status (students vs others)	*0.08*	*0.01*	*0.05*	*0.12*	*0.14*
	ΔR2 for both interaction effects	*0.05***	*0.04**	*0.02*	*0.03**	*0.03**

PGWBI=Personal General Well-Being Index

N = 298. Interaction coefficients and percentage of variance explained by them are reported in italics.
° $p < 0.07$; * $p < 0.05$; ** $p < 0.01$; *** $p < 0.001$.

Table S2. Partially standardized regression coefficients and percentage of variance explained for joint moderation effects of Gender and Employment status on the association between Family resilience at T1 and PGWBI dimensions at T2, when controlling for their baseline values – Study 2.

Moderator		Anxiety	Depressed mood	Positive well-being	Self-control	Vitality
Males	Full-time workers	-0.14	0.28	0.69**	0.14	0.34
	Part-time workers	0.39	10.34***	10.32***	0.66*	0.71*
	Not working students	0.12	0.94***	0.93***	0.47	0.79**
Females	Full-time workers	-0.08	-0.41	-0.05	-0.4	-0.13
	Part-time workers	0.45*	0.65***	0.59**	0.57**	0.24
	Not working students	0.18	0.25	0.26**	0.38**	0.32*
Interaction coefficient for gender		*0.00*	*-0.69***	*-0.74***	*-0.09*	*0.03*
Interaction coefficients for employment status (part-time workers vs others)		*0.53*	*10.06****	*0.63**	*0.52°*	*0.38*
Interaction coefficients for employment status (students vs others)		*0.26*	*0.65***	*0.24*	*0.33*	*0.45*
ΔR2 for both interaction effects		*0.04*	*0.13***	*0.09**	*0.03*	*0.04*

PGWBI=Personal General Well-Being Index

N = 101. Interaction coefficients and percentage of variance explained by them are reported in italics.

° $p < 0.07$; * $p < 0.05$; ** $p < 0.01$; *** $p < 0.001$.
